# Exploration of the Processes of “Iranian Journal of Public Health” during 2016–2019

**Published:** 2020-02

**Authors:** Dariush D FARHUD

In order to have a bird’s eye view on the output of **Iran J Public Health**, hereby this analysis is presented for the year 2019. Besides, this editorial will compare the trend of whole publication process during 2016–2019.

**Fig. 1:**
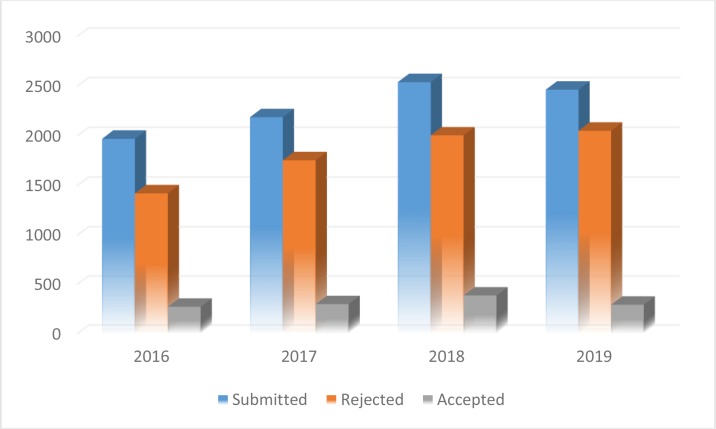
Total number of articles submitted during 2016–19 in the context of the frequency of submission, rejection and acceptance rate.

The total number of manuscripts received during 2019 was 2464 from 52 countries. Of course, only the country of corresponding author was considered, so altogether much more countries we had in the panel. Again, Iran had the highest rate of submission, followed by China and Turkey (Table 1). Figure 1 presents total number of articles published during 2016–19 in the context of the frequency of submission, rejection and acceptance rate.

**Table 1:** Frequency of manuscripts received by Iran J Public Health during 2018 in terms of the frequency of submission, rejection and acceptance rate

**No.**	**Country**	**Submission**	**Reject**	**Accepted**
**1**	**Albania**	2	2	0
**2**	**Argentina**	1	0	0
**3**	**Armenia**	4	3	1
**4**	**Bangladesh**	8	3	1
**5**	**Brazil**	13	3	4
**6**	**Bulgaria**	12	3	5
**7**	**Czech**	1	0	0
**8**	**China**	360	267	67
**9**	**Colombia**	2	1	0
**10**	**Egypt**	14	12	1
**11**	**Ethiopia**	3	3	0
**12**	**Georgia**	1	0	0
**13**	**Greece**	4	4	0
**14**	**Hungary**	1	0	0
**15**	**India**	29	29	0
**16**	**Indonesia**	134	121	7
**17**	**Iran**	1197	1051	106
**18**	**Iraq**	44	42	1
**19**	**Italy**	2	1	1
**20**	**Japan**	7	3	4
**21**	**Jordan**	6	6	0
**22**	**Kazakhstan**	20	13	5
**23**	**Korea**	116	52	52
**24**	**Kosova**	2	2	0
**25**	**Macedonia**	2	0	0
**26**	**Malaysia**	35	29	3
**27**	**Mexico**	5	5	0
**28**	**Mongolia**	1	1	0
**29**	**Montenegro**	3	1	2
**30**	**Morocco**	9	9	0
**31**	**Nepal**	1	1	0
**32**	**Nigeria**	10	8	2
**33**	**Omman**	1	1	0
**34**	**Pakistan**	82	74	7
**35**	**Palestinian**	2	2	0
**36**	**Poland**	10	9	1
**37**	**Qatar**	2	2	0
**38**	**Romania**	17	12	5
**39**	**Russia**	4	3	1
**40**	**Saudi Arabia**	6	6	0
**41**	**Serbia**	29	22	7
**42**	**Slovakia**	8	5	2
**43**	**South Africa**	1	1	0
**44**	**Taiwan**	7	6	1
**45**	**Thailand**	15	11	2
**46**	**Tunisia**	3	3	0
**47**	**Turkey**	215	206	5
**48**	**UK**	1	1	0
**49**	**Ukraine**	5	5	0
**50**	**United States**	1	1	0
**51**	**Vietnam**	5	5	0
**52**	**Zambia**	1	1	0
**Total**	**-**	2464	2051	293

**Note:** Some manuscripts submitted during 2019, are still in the process of peer review so we have no idea of their destination

Out of total submission of 2464 articles during 2019, 2051 articles were rejected after initial in-house evaluation in addition to subsequent peer review. Therefore the acceptance rate for this year was 11.9%.

In 2019, the rate of plagiarism cases fortunately was decreased significantly. It might be due to policy of the journal and restrictions imposed by Editorial Board based on the COPE rules. Still, authors of minor cases of plagiarism are given a chance to amend their manuscripts precisely but major cases are rejected.

**Fig. 2:**
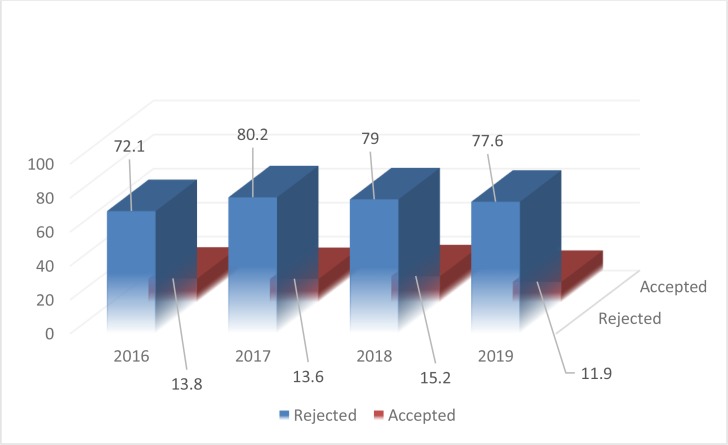
The percentage of acceptance and rejection rate during 2016–19.

The Journal continued its policy as for exact peer review rules including in-house evaluation followed by double blind peer review system. The reasons for rejecting a manuscript during in-house evaluation are various but the most important cases are out of scope cases, poor outcome, local studies, clinical contents etc. Figure 2, demonstrates the total number of articles published during 2016–2019 in terms of the percentage of acceptance and rejection rate. It is worth mentioning that some manuscripts submitted during 2019, are still in the process of peer review so we have no idea of their destination. However, the rejection rate in 2019 was 83.2%.

**Fig. 3:**
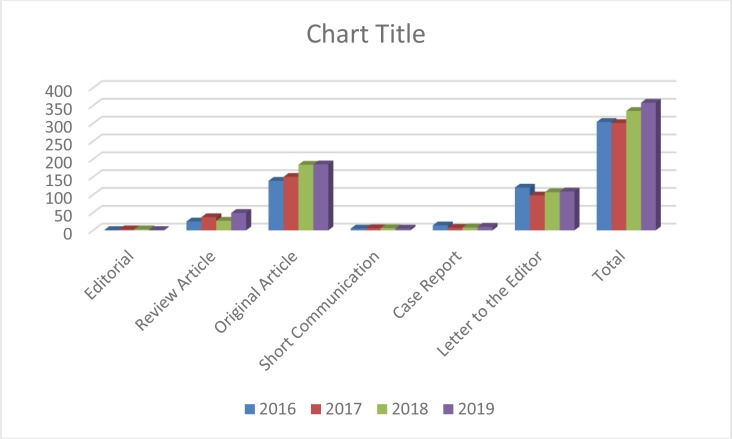
Total number of articles during 2016–19 based on the type of published papers.

The types of articles published during 2016–2019 are shown in Fig. 3. Accordingly, Original Articles had the highest rate of publication during the last four years.

Due to high flow of submitted manuscripts, in many cases, the authors were requested to change the format of “Original Article” to “Letter to the Editor”.

The good news for us and respected authors was that the Impact Factor announced by Clarivate Analytics (ISI) for the year 2019 was 1.225 and Eigen factor Score as 0.003740. Obviously it is increased year by year and we do hope to maintain this increasement.

**Fig. 4:**
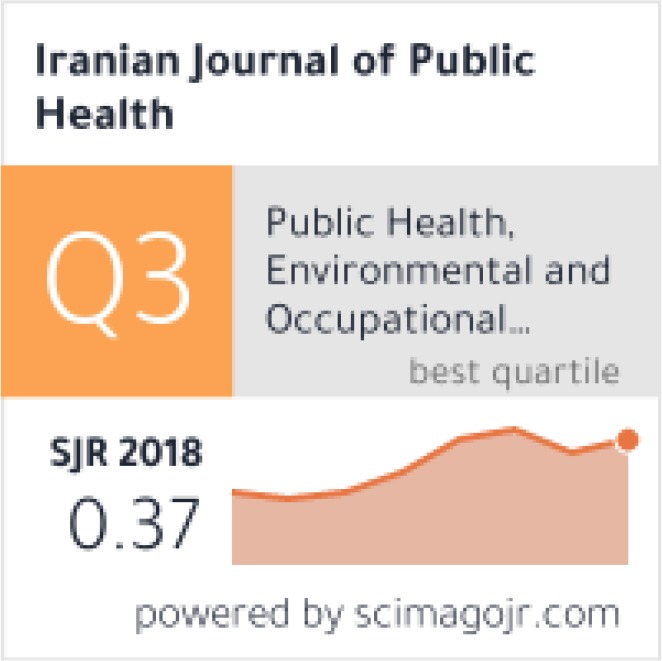
Key Indicators announced by Scimagojr.com for the year 2018.

According to http://www.scimagojr.com/, the H index of the journal was 27 until 2018, moreover the rank of the journal was Q3 (Fig. 4). Besides, SCOPUS has reported the Site Score of the journal as 0.93 for 2018, besides SNIP as 0.809 as well SJR as 0.368. All these rates shows the improvement of the quality of the journal.

